# Novel redox-active enzymes for ligninolytic applications revealed from multiomics analyses of *Peniophora sp*. CBMAI 1063, a laccase hyper-producer strain

**DOI:** 10.1038/s41598-019-53608-1

**Published:** 2019-11-26

**Authors:** Lívia B. Brenelli, Gabriela F. Persinoti, João Paulo L. Franco Cairo, Marcelo V. Liberato, Thiago Augusto Gonçalves, Igor V. R. Otero, Pedro H. Mainardi, Claus Felby, Lara D. Sette, Fabio M. Squina

**Affiliations:** 1 Brazilian Biorenewables National Laboratory (LNBR), Brazilian Center for Research in Energy and Materials (CNPEM), Campinas, Brazil; 2 Programa de Processos Tecnológicos e Ambientais, Universidade de Sorocaba (UNISO), Sorocaba, Brazil; 3 Universidade Estadual Paulista (UNESP), Instituto de Biociências, Rio Claro, Brazil; 4 Departamento de Bioquímica e Biologia Tecidual, Instituto de Biologia, Universidade de Campinas (UNICAMP), Campinas, SP Brasil; 5 University of Copenhagen, Faculty of Science, Department of Geosciences and Natural Resource Management, Frederiksberg C, Denmark

**Keywords:** Enzymes, Industrial microbiology

## Abstract

The repertoire of redox-active enzymes produced by the marine fungus *Peniophora* sp. CBMAI 1063, a laccase hyper-producer strain, was characterized by omics analyses. The genome revealed 309 Carbohydrate-Active Enzymes (CAZymes) genes, including 48 predicted genes related to the modification and degradation of lignin, whith 303 being transcribed under cultivation in optimized saline conditions for laccase production. The secretome confirmed that the fungus can produce a versatile ligninolytic enzyme cocktail. It secretes 56 CAZymes, including 11 oxidative enzymes classified as members of auxiliary activity families (AAs), comprising two laccases, Pnh_Lac1 and Pnh_Lac2, the first is the major secretory protein of the fungi. The Pnh_Lac1-mediator system was able to promote the depolymerization of lignin fragments and polymeric lignin removal from pretreated sugarcane bagasse, confirming viability of this fungus enzymatic system for lignocellulose-based bioproducts applications.

## Introduction

Marine-derived fungal species have been considered attractive producers of ligninolytic, hemicellulolytic and other industrial enzymes, presenting different properties compared to terrestrial enzymes, such as high salt tolerance and thermostability^1^. The white-rot basidiomycete *Peniophora* sp. CBMAI 1063, isolated from the seawater sponge *Amphimedon viridis*, has been reported as a producer of oxidative enzymes under saline and non-saline conditions, and recently a transcriptome analysis revealed several sequences encoding for putative laccases^2,3^.

Fungi from the Basidiomycota phylum are considered the most efficient lignin degraders, and according to their lifestyle and ability to degrade polymeric constituents, they can be classified as white-rot or brown-rot fungi^4^. White-rot fungi simultaneously attack lignin, hemicellulose and cellulose, the main components of the plant cell wall, and their genomes generally have more genes encoding for oxidative enzymes when compared to brown-rot and other groups of basidiomycetes^5,6^. Lignin degradation/modification in white-rot fungi is generally performed via the action of enzymes such as laccases and peroxidases while in brown-rot fungi it is driven by Fenton reactions (Fe^2+^ + H_2_O_2_ → Fe^3+^ + HO^•^ + OH^−^ and Fe^3+^ + H_2_O_2_ → Fe^2+^ + HOO• + H^+^). Laccase and peroxidases genes are abundant in white-rot fungi genomes and reduced or absent in brown-rot fungi^5,7^.

The degradation and modification of aromatic compounds by white-rot fungi involves several enzymes classified as Auxiliary Activity (AA) families in the CAZy database^7^. The AA1 family contains laccases, which can be classified in three subfamilies AA1_1, AA1_2 and AA1_3. The subfamily AA1_1 has members of the ‘true’ laccases EC 1.10.3.2 or the blue copper oxidases, which are able to oxidize a wide range of aromatic compounds, including lignin, with an oxygen molecule as the final electron acceptor^8^. The AA2 family includes lignin peroxidases, manganese peroxidases and versatile peroxidases, which all use hydrogen peroxide as a cofactor for lignin degradation^7^. Moreover, enzymes from families AA4 (vanillyl-alcohol oxidases), AA5 (glyoxal oxidases/alcohol oxidases) and AA6 (1,4-benzoquinone reductases) are also correlated with the oxidation or reduction of phenolic compounds derived from lignin^9,10^. Other AA families are also reported to potentially drive lignin degradation and modification through Fenton reactions, such as AA3, AA7 and AA8, since the majority of their members can generate H_2_O_2_ as a by-product^7,11^.

In this report, we unveiled the repertoire of genes encoding for ligninolytic enzymes from the genome of *Peniophora* sp. CBMAI 1063, allied with the transcriptome and secretome analyses of the fungus growth in an optimized cultivation media for laccase production, which was formulated without any complex lignocellulosic component. Although the omics analyses were focused on the *Peniophora* sp. CBMAI 1063 oxidative enzyme system, cellulose and hemicellulose-degrading enzymes were identified from the secretome analysis as well. Furthermore, the major laccase secreted by *Peniophora* sp. CBMAI 1063 was identified by mass spectrometry. Structural characteristics of the protein and its potential application to promote lignin modification and degradation were explored.

## Material and Methods

### Microorganism

The marine-derived fungus *Peniophora* sp. was isolated from the Brazilian sponge *A. viridis*, collected from a coral reef in São Sebastião, São Paulo State, Brazil^12^. The strain was deposited at the Brazilian Collection of Microorganisms from the Environment and Industry—CBMAI, under accession number CBMAI 1063.

### Fungal growth

Solid cultivations of *Peniophora* sp. CBMAI 1063 were carried out in Petri dishes containing malt extract (20 g L^−1^) and agar-agar (15 g L^−1^) at 28 °C. After 7 days of growth, six agar-plugs measuring approximately 0.9 cm in diameter were inoculated in non-baffled 125 mL Erlenmeyer flasks containing 50 mL of an optimized medium which includes yeast extract (2.0 g L^−1^), peptone extract from casein (2.7 g L^−1^), malt extract powder (1.4 g L^−1^) and glucose (2.7 g L^−1^). The reagents were dissolved in distilled water with 65% (v/v) of adapted artificial seawater, containing: MgCl_2_ (10.83 g L^−1^), CaCl_2_ (1.51 g L^−1^), SrCl_2_ (0.02 g L^−1^), NaCl (23.9 g L^−1^), Na_2_SO_4_ (4,0 g L^−1^), KCl (0.68 g L^−1^), NaHCO_3_ (0.2 g L^−1^), KBr (0.1 g L^−1^) and H_3_BO_3_ (0.03 g L^−1^)^2^. The culture medium was supplemented with aqueous CuSO_4_ to a final copper concentration of 2 mM. The flasks were maintained at 28 °C under constant agitation (140 rpm) for 7 days. Contents of the inoculated flasks was used as inoculum in a Fermac320 bioreactor (Electrolab Limited, UK) with 6.4 L total volume at an equivalent proportion of 8.5% (v/v) for 5 days. The bioreactor was placed in a temperature-controlled room at 20 °C and air filtered through two 0.2 µm filters (Sartorius-GER) was injected to the system via an “L” type sparger with 5 bottom holes^2^.

### DNA extraction and sequencing

Genomic DNA was extracted from the bioreactor culture using the cetyltrimethyl ammonium bromide method adapted from the literature^13,14^, and purified using a Power Clean^®^ DNA Clean-Up Kit (Mo Bio Laboratories). A sequencing library was built from high-quality genomic DNA using the Nextera DNA library preparation Kit (Illumina) with fragment size around 300 pb. Next, the library was submitted to sequencing on an Illumina Hiseq 2500 instrument with the HiSeq Rapid kit v2 chemistry in paired-end mode (2 × 100 bp), at the NGS sequencing facility of the Brazilian Biorenewables National Laboratory (LNBR/CNPEM).

### Genome assembly

The genome of *Peniophora* sp. CBMAI 1063 was sequenced and generated 55 million paired-end reads (2 × 100 bp). Reads were processed with Trimmomatic 0.32^15^ to remove low quality reads and adapter sequences, resulting in 39 million quality-filtered reads. The genome size was estimated based on k-mer count statistics (Kmergenie). Before assembly, reads were normalized based on k-mer abundance with Khmer^16,17^ to a coverage of 20X and subjected to *de novo* assembly using Velvet version 1.2.10^18^ with k = 55. The complete subset of quality filtered reads was used to improve the assembly and for scaffolding using Pilon version 1.16^19^ and SSPACE version 3.0^20^, respectively.

### Gene prediction and annotation

A transcriptome analysis of *Peniophora* sp. CBMAI 1063 cultivated for 7 days at 28 °C in Erlenmeyer flasks was previously reported by Otero *et al*.^3^ and the raw sequence data, available in the Short Read Archives (SRA) GenBank database (accession number SRR5799684), was used in the present study as evidence for gene prediction and to determine the expression profile of the CAZymes. In order to use RNA-seq data for gene prediction, QC reads were aligned to the scaffolds produced by SSPACE using Hisat2^21^ and subjected to the BRAKER1 v1.9^22^ an automated genome annotation pipeline, which uses RNA-seq spliced alignments as evidence for GeneMark-ET and Augustus gene structure predictions^22^. “Non-coding rRNA genes were identified using both ITSx^23^ version 1.0.11 and RNAmmer^24^ version 1.2, and tRNA genes were annotated using tRNAscan^25^ version 1.3.1. Completeness of the genome gene set was estimated using the Benchmarking Universal Single-Copy Orthologs (BUSCO)^26^ version 1.1. Functional annotation was performed by comparison of the predicted protein sequences in the SwissProt database^27^ UniRef90 database^28^, PFAM^29^, Hidden Markov models (HMM) available at dbCAN 2.0 (automated carbohydrate-active enzyme annotation)^30^ and EggNOG (evolutionary genealogy of genes with enhanced non-supervised orthologous groups) databases^31^.

### Phylogenetic analysis

Phylogenetic analysis was conducted using 36 organisms from the Basidiomycota phylum with public available genome sequences at JGI and the genome sequence of *Peniophora* sp. CBMAI 1063. Among the genomes analyzed, 35 are from the Agaricomycetes class and 2 from Ustilaginomycotina, which was used as the out-group. BUSCO was used to search for conserved genes among the 37 genomes analyzed. A set of 92 single copy gene markers present in all species was used to perform the phylogenetic analysis. The protein sequences of each marker gene were aligned using MAFFT version v7.299b^32^ and concatenated into a super matrix using FASconCAT-G version 1.2. The phylogenetic inference was performed using RAxML version 8.2.0^33^ with PROTGAMMAWAG model.

### Secretome analysis

The crude fungal extract from one growth culture batch in the bioreactor was centrifuged at 5,000 *g* for 30 minutes and submitted to electrophoresis analysis in polyacrylamide gel containing 0.1% (w/v) sodium dodecyl sulfate (SDS-PAGE). The gel-bands were excised from the SDS-PAGE and protein digestion for the mass spectrometry-based analyses was performed in two steps over two days. Firstly, SDS and Coomassie stain were removed with 500 µL of destaining solution (10% glacial acetic acid and 10% ethanol) for 2 h. The bands were dehydrated for 5 min with 200 µL acetonitrile, reduced for 30 min with 30 µL dithiothreitol solution (0.01 mol L^−1^) and alkylated for 30 min with 30 µL iodoacetamide solution (0.05 mol L^−1^) and washed with ammonium bicarbonate solution (0.1 mol L^−1^) for 10 min. A second dehydration step was performed with acetonitrile, followed by rehydration with sodium bicarbonate solution (0.05 mol L^−1^). Proteins were digested with 30 µL of trypsin (1.0 mg mL^−1^) in ammonium bicarbonate (0.05 mol L^−1^) at 37 °C for 12 h. Then, 20 µL of extraction solution composed of 5% (v/v) formic acid was added to each microtube and incubated for 10 min at room temperature. After centrifugation, the supernatant was transferred to another microtube and 30 µL of extraction solution composed of 5% (v/v) formic acid in 50% (v/v) acetonitrile were added. The supernatant was transferred to a tube containing the extract from the previous step after 10 min. The last procedure was repeated, and the samples were evaporated under vacuum to approximately 1 µL final volume. The samples were stored at −20 °C until further analysis by LC–MS/MS. Each sample was mixed with 12 μL of 0.1% (v/v) formic acid, and 4.5 μL of the peptide mixture were injected into the liquid chromatography-tandem mass spectrometry (LC–MS/MS) chromatograph (RP-nanoUPLC, nanoAcquity, Waters, Milford, MA). Peptide separations were performed in a C18 column (100 nm × 100 mm) previously equilibrated with a 0.1% (v/v) formic acid buffer. The elution gradient ranged from 2 to 90% (v/v) acetonitrile in 0.1% (v/v) formic acid at 0.6 μL min^−1^. Eluted peptides were analyzed in a quadrupole time of flight (Q-TOF) spectrometer (Ultima Mass Spectrometer, Waters Milford, MA) operating in the “top three-MS and MS/MS mode” (Ultima Mass Spectrometer, Waters software). Spectra were acquired using the Mass Lynx v.4.1 software (Waters, Milford, MA, USA), and the raw data were converted to “peak list format (mgf)” using the Mascot Distiller software v.2.3.02, 2009 (Matrix Science Ltd., London, UK). Results were processed using the Mascot v.2.3.02 engine software (Matrix Science Ltd.) against the *Peniophora* sp. CBMAI 1063 genome sequence database generated in this work. The following parameters were used in this process: carbamidomethylation as a fixed modification, oxidation of methionine as a variable modification, one trypsin cleavage error and a maximum allowable peptide mass error of 0.1 Da. The resulting Mascot data were analyzed for protein identification using Scaffold 3.5.1 (Proteome Software, Portland, OR). The defined parameters were as follows: minimum protein probability of 80%, minimum peptide probability of 90% and uniquely different minimum peptide of 1. Proteins with scores up to 10% false discovery rate (FDR) for a protein and 5% FDR for a peptide were accepted. The presence of a signal peptide on identified proteins was predicted by SignalP v.4.0 (http://www.cbs.dtu.dk/services/SignalP/)^34^ and the subcellular localization of proteins was predicted by YLoc (abi.inf.unituebingen.de/Services/YLoc/webloc.cgi)^35^.

### Laccase production and purification

Laccase production and purification by ion exchange chromatography followed by size exclusion chromatography were performed according Mainardi *et al*.^2^. The fractions corresponding to the two intense peaks signal detected at 260 and 280 nm were concentrated individually and submitted to electrophoresis analysis in polyacrylamide gel containing 0.1% (w/v) SDS-PAGE. The gel-bands were excised from the SDS-PAGE and laccases identities were confirmed in duplicate by mass spectrometry as described above with FDR of 1.35%; and protein concentration was determined by the Bradford method^36^.

### Laccase 3D-structure prediction by homology modeling

The three-dimensional structure of the major secreted laccase was generated by homology modeling on the I-TASSER server^37^. The laccase amino acid sequence was determined by mass spectrometry analysis; and the final model was generated based on the crystallographic structure of five different laccases from the AA1_1 family (PDBid: 1A65, 1V10, 2QT6, 5DAO and 5E9N) and one unclassified laccase (PDBid: 2HRG).

### Laccase activity assay

Laccase activity was determined using syringaldazine (SGD) as the substrate as described in Mainardi *et al*.^2^. Oxidation of SGD was monitored at 525 nm for 5 minutes (readings every 20 seconds) at room temperature in a Tecan^®^ Infinite spectrophotometer. One unit of enzyme activity was defined as the amount of enzyme required to oxidize 1 µmol of SGD per minute.

### Laccase-Mediator system (LMS) assays for lignin modification

The alkali-lignin used in this study (96% purity) was extracted from steam-exploded sugarcane bagasse according Brenelli *et al*.^38^. Initially, a lignin stock solution at 10 mg mL^−1^ was prepared in NaOH 0.1 mol L^−1^. In the reaction system, 100 µL of the lignin stock solution was incubated with sodium acetate buffer 0.05 M at pH 5.0, purified laccase at 0.6 U g lignin^−1^ and 2,2′-Azino-bis (3-ethylbenzothiazoline-6-sulfonic acid) diammonium salt (ABTS) (Sigma-Aldrich^®^) as a mediator at a final concentration of 1 mmol L^−1^. The reaction (1.0 mL final volume) was incubated at 30 °C and 1000 rpm shaking in a Thermomixer (Eppendorf^®^) for 72 h. Incubations containing inactivated laccase and the ABTS mediator were used as controls. After incubation, the laccase activity was stopped by adding NaN_3_ at a final concentration of 0.05% (w V^−1^)^39^. The reaction mixture was centrifuged (12,000 x *g* for 10 min at 4 °C) and the supernatant collected for subsequent analysis. Changes in the molecular weight distribution of the soluble lignin and lignin-derived compounds were detected by gel permeation chromatography (GPC) using a Superdex 30 column (65 cm × 1.6 cm) in an automated AKTA^TM^ Purifier system (GE Healthcare) equipped with a UV detector (280 nm) and NaOH 0.1 mol L^−1^ as the eluent. The flow was 0.1 mL min^−1^ at room temperature with 500 µL sample injection volume. Tannic acid, ferulic acid, coumaric acid, cinnamic acid, hydroquinone and vanillin from Sigma Aldrich^®^ and lignins with known molecular weight were used as internal standards. A UV-vis spectroscopy analysis was performed in a Tecan^®^ Infinite spectrophotometer. The samples were diluted 20-fold in NaOH 0.1 mol L^−1^ and the UV-vis absorption spectrum (220 to 500 nm) was recorded using a 1 cm quartz cuvette.

### LMS-based pretreatment

The pretreated sugarcane bagasse (SCB) used in this study was obtained by steam explosion (190 °C, 1.3 MPa, 15 min) as described previously^40^. The chemical composition of SCB was determined using the NREL method^41^ for lignocellulosic biomasses and was reported to be 52% glucan, 6% xylan, 24% lignin, 0.2% galactan and 0.5% arabinan. The commercial cellulolytic cocktails employed in this study were Accellerase® (DuPont) and Cellic^®^ Ctec2 (Novozymes A/S, Bagsvaerd, Denmark). Cellulase activities of the enzymatic preparations measured via the filter paper assay were 168 and 250 filter paper units (FPU) mL^−1^, respectively. Both preparations were stored at 4 °C until performing the enzymatic hydrolysis assay. First, LMS assays were performed in 2.0 mL tubes with 2.5% (w V^−1^) dry matter (DM) in sodium acetate buffer pH 5.0; the reaction volume was 1 mL and it was maintained at 30 °C and 1000 rpm shaking in a Thermomixer^®^ (Eppendorf). Purified laccase enzymes were added at a dosage of 0.02 U g biomass^−1^ as well as the ABTS mediator to a final concentration of 1 mmol L^−1^. Assays containing laccase inactivated by boiling and the ABTS mediator were used as controls. After 72 h the laccase activity was stopped by adding sodium azide (NaN_3_) at a final concentration of 0.05% (w V^−1^), then the commercial cocktails Accellerase^®^ or Cellic^®^ CTec2 were added in a low dosage of 2.5 FPU g cellulose^−1^ and incubated for 72 h (50 °C, 1000 rpm) in a ThermoMixer^®^ (Eppendorf). The supernatant was separated by centrifugation and filtered (0.45 µM, Millipore^®^). Reducing sugars were measured by reacting 100 µL of the supernatant with 100 µL of 3,5-dinitrosalicylic acid for 5 min at 99 °C^42^. The cooled solution was analyzed at 540 nm in an Infinite M200^®^ spectrophotometer (Tecan-Switzerland) and compared with an internal calibration curve using glucose. All enzymatic hydrolysis assays were carried out in triplicate and the average and standard deviation values were determined. The supernatant from non-hydrolyzed LMS-treated SCB was analyzed by GPC as described previously^38^.

### Statistical analysis

A statistical analysis was performed by analysis of variance (One-way ANOVA) with a probability level (P) less than 5% (P < 0.05), using the program STATISTICA 5.5 from StatSoft Inc. (Tulsa, OK, USA).

## Results and Discussion

### The *Peniophora* sp. CBMAI 1063 genome content is distinct to other related species

The genome of the marine-derived *Peniophora* sp. CBMAI 1063 was sequenced using Illumina sequencing, with 165X coverage and reaching 93% genome completeness. The resulting draft genome assembly of *Peniophora* sp. CBMAI 1063, access number PRJEB28379, is 47.9 Mb in length with an N50 of 155.8 Kb, and average G + C content of 55% (Table S1). The genome size is similar to the other two *Peniophora* genomes available on the JGI Mycocosm portal: 48.4 Mb for *Peniophora* sp. CONTA (Lopni1) and 46.0 Mb for *Peniophora* aff. cinerea (Ricme1), both of which are plant pathogens.

**Figure 1:**
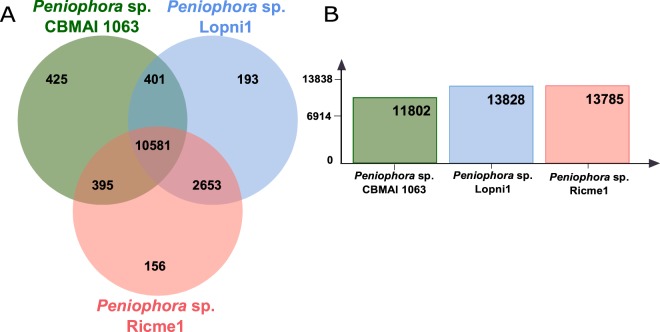
A Venn diagram showing the distribution of orthologous gene clusters across the marine-derived *Peniophora* sp. CBMAI 1063, *Peniophora* sp. CONTA (Lopni1) and *Peniophora* aff. cinerea (Ricme1) (**A**) and the total number of orthologous gene clusters of each organism (**B**).

**Figure 2:**
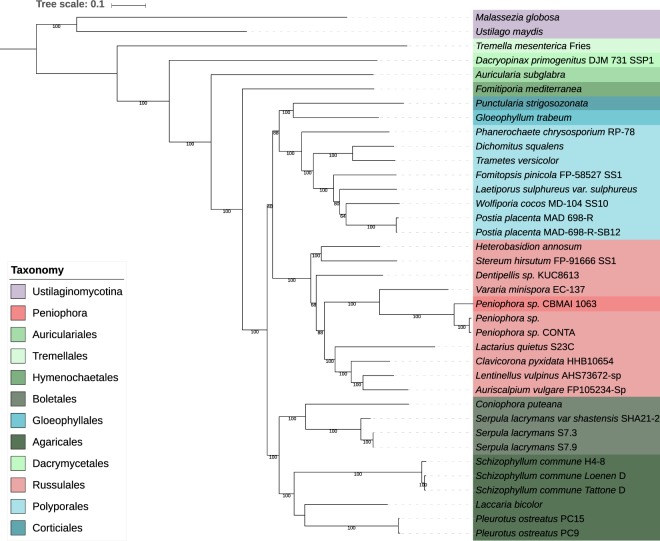
Phylogenetic tree of the Agaricomycetes class based on 34 fungi genomes distributed among the Basidiomycota phylum. A set of 92 single copy gene markers was used to perform the phylogenetic analysis. Bootstrap values for 1000 replicates are show in the branches.

The majority of the predicted genes are common among the three strains analyzed, however a significant number of genes were found to be specific to each strain (Fig. 1). Among these genes, 457 were found to be specific to CBMAI 1063, 187 to Lopni1, and 164 to Ricme1 strains. The two plant-pathogen fungi strains, Lopni1 and Ricme1, shared 2791 orthologous genes (Fig. 1). The number of orthologous genes shared between *Peniophora* sp. CBMAI 1063 with Lopni1 or Ricme1 is significantly lower, only 370 and 381, respectively. The phylogenetic analysis using a set of 92 single-copy gene markers and 37 genomes from Basidiomycetes phylum (Fig. 2) indicated that *Peniophora* sp. CBMAI 1063 clustered together with the other two *Peniophora* species previously sequenced by JGI, forming a monophyletic clade in the Russulales order.

### The *Peniophora* sp. CBMAI 1063 ligninolytic enzyme content

**Figure 3:**
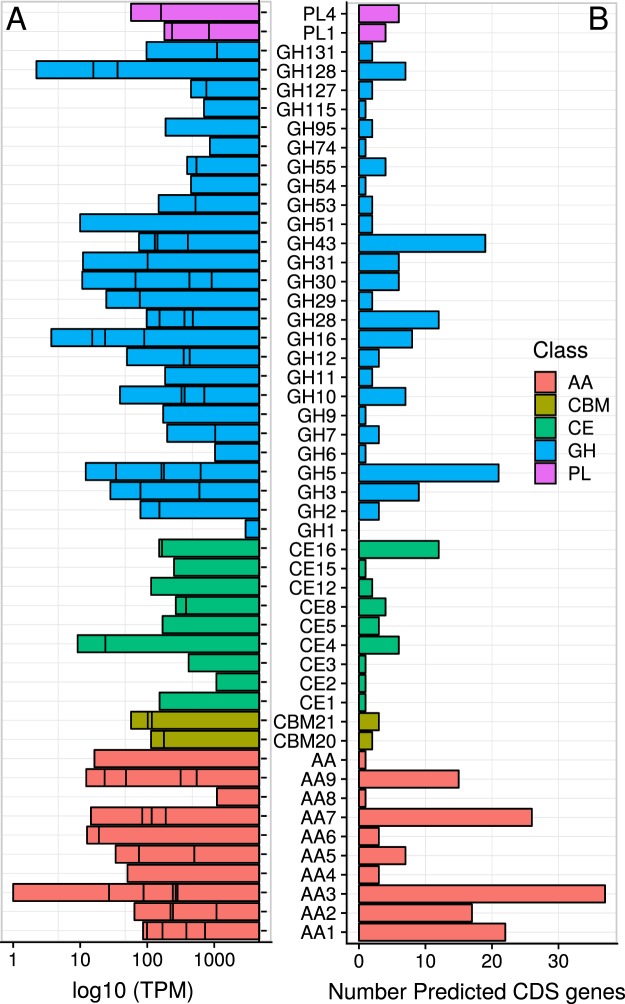
Transcriptome and genome profile of the marine-derived *Peniophora* sp. CBMAI 1063. (**A**) Transcriptome profile represented as log_10_ of TPM (Transcripts Per Million). (**B**) Genome profile represented as the number of predicted genes encoding CAZymes among the different classes of enzymes according to the CAZy database (http://www.cazy.org/).

The genome of *Peniophora* sp. CBMAI 1063 encodes 17,714 predicted/putative genes, including 11,827 clusters of orthologous genes. A comparison of the predicted genes against the Carbohydrate-Active Enzymes database^7^ (CAZy) identified 310 predicted coding genes related to ligninolytic and carbohydrate-active enzymes (Fig. 3). Among the predicted genes encoding for the degradation/modification of lignin and the derived phenolic compounds, *Peniophora* sp. CBMAI 1063 exhibited 18 genes from family AA1 (laccases), 17 genes from AA2 (peroxidases), 3 genes from AA4 (vanillyl-alcohol oxidases), 7 genes from AA5 (glyoxal and alchool oxidases) and 3 from AA6 (1,4-benzoquinone reductase). Among those genes, 17 laccases AA1, 16 peroxidases AA2 and 7 glyoxal/alcohol oxidases AA5 are predicted to possess a secretion signal while all genes of AA4 and AA6 lack this signal.

A total of 41 coding genes of protein family AA3 (glucose oxidase, glucose dehydrogenases and cellobiose dehydrogenases) along with 16 genes of AA9 (LPMOs), 27 genes of AA7 (glucooligosaccharide oxidases) and 1 gene of AA8 (cellobiose dehydrogenases) were present in the genome of *Peniophora* sp. CBMAI 1063 (Fig. 3). The LPMOs from family AA9 have been found only in fungi and they are often co-expressed with sugar oxidases, such as those from the families AA3, AA7 and AA8^43^. All AA9 genes exhibit secretion signals while for the AA3-coding genes the signal peptide is absent.

Furthermore, several genes encoding glycoside hydrolases (GH) were identified, including cellobiohydrolases from families GH6 and GH7, xylanases from GH10 and GH11, and pectinases from family GH28. Protein-coding genes for carbohydrate esterases (CE) families CE4, CE8 and CE16, and CBM families 20 and 21 (Fig. 3) were also identified. A high number of putative genes encoding for family GH5 (21 genes) and GH43 (19 genes), whose characterized members are typically endoglucanases and xylosidases, respectively, were found in the *Peniophora* sp. CBMAI 1063 repertoire of CAZymes. Interestingly, the fungus contains putative/predicted GH9-encoding genes, a family of endoglucanases found in anaerobic bacteria producing cellulosomes, plants and termites^44^.

The lignocellulolytic capabilities of marine fungi associated with algae, sponges, and mangrove habitats have previously been highlighted in other studies^1,45^, although the number of available sequenced genomes is still restricted. The high content of lignocellulosic materials from terrestrial sources that enter the ocean, as well as the symbiotic relationships with other organisms, justify the presence of genes encoding putative CAZymes in marine fungi, which may be found in high number compared to their terrestrial plant-degrading counterparts^46^.

Furthermore, the oceans are the largest source of biogenic organohalogens containing chlorine or bromine, which are biosynthesized by myriad seaweeds, sponges, corals, tunicates, bacteria, and other marine life^47^. In particular, the function of organohalogens in sponges is presumably to prevent feeding by fish and fouling by barnacles, bacteria, and fungi^48^. Since pyrroles, indoles, phenols, and tyrosines are commonly found to be halogenated in sponges, it is not a surprise that associated bacteria, microalgae or fungi are adapted to biosynthesize specific metabolites^49^. This work does not intend to elucidate fungal-sponge relationships by genomic comparisons, however this wide repertoire of CAZymes, in particular oxidoreductive enzymes, may be important to enable *Peniophora* sp. CBMAI 1063 to live in close association with its sponge host (*Amphimedon viridis)* in a marine environment.

### Transcript encoding extracellular AA family members were expressed by *Peniophora* sp. CBMAI 1063 during growth in optimized conditions

Otero *et al*.^3^ described a preliminary global transcriptome analysis of *Peniophora* sp. CBMAI 1063 cultivated in a medium optimized for laccase production for 7 days in Erlenmeyer flask. Herein, the former RNA-Seq data set was used to validate the genome analysis, and also to depict the set of other partner genes encoding for lignin degradation and modifications enzymes. Additionally, the transcriptome data assisted in understanding not only the ligninolytic system, but also the cellulolytic genes co-expressed in media, which was formulated without a complex carbon source, i.e. lignocellulosic biomass.

From the 310 predicted genes related to lignin modification and degradation and CAZymes, 303 were expressed in the optimized media (Fig. 3). The genes encoding ligninolytic enzymes from families AA1, AA2, AA4, AA5 and AA6 were abundant in the transcriptome (Fig. 3). *Peniophora* sp. CBMAI 1063 exhibited high laccase activity in the optimized cultivation media^1,2^, corroborating with the present analyses, which identified the complete set of 18 genes expressed as predicted for the family AA1 (Fig. 3).

The AA1 family includes multicopper oxidases, including laccases, ferroxidases and laccase-like multicopper oxidases. Among all genes predicted as AA1 in the fungus, 15721.t1, g1591.t1, and g714.t1 were the most abundant transcripts found, considering the TPM of 53.2, 46.2 and 26.7, respectively. Concerning the family AA2, the genes encoding for peroxidases g10529.t1, g14863.t1, and g8820.t1 were the most abundant (Fig. 3). The gene of highest TPM among all CAZymes was the AA3 gene (g15979.t1). This transcript encodes a cytoplasmatic protein containing a glucose-methanol-choline (GMC) oxidoreductase domain, which is related to hydrogen peroxide generation and may act as a co-factor for peroxidases^50^. LPMOs from family AA9 and their electron donor protein partners, as well as the genes coding for AA3, AA7, and AA8, were also present in the transcriptome. Among the GH families involved in cellulose, hemicellulose and pectin degradation, genes coding for the families GH1, GH3, GH5, GH7, GH10, GH11, GH43 and GH51 were identified along with the transcription of CE genes from several families (CE4, CE12, CE5, CE16) (Fig. 3).

### Secretomic analysis

A wide variety of oxidoreductases and CAZymes related to the degradation of plant cell wall polymers are present in Basidiomycota species^51^. However, depending on the species and lifestyles, the repertoire of enzymes and their gene numbers can differ significantly^52^.

In this work the *Peniophora* sp. CBMAI 1063 secretome was obtained when cultivated under optimized conditions for laccase production in bioreactor^2^. Additionally, the FDR based on use of a randomized decoy was 0.03%, indicating that the database employed was of high quality for mass spectrometry-based proteomics analysis.

**Figure 4:**
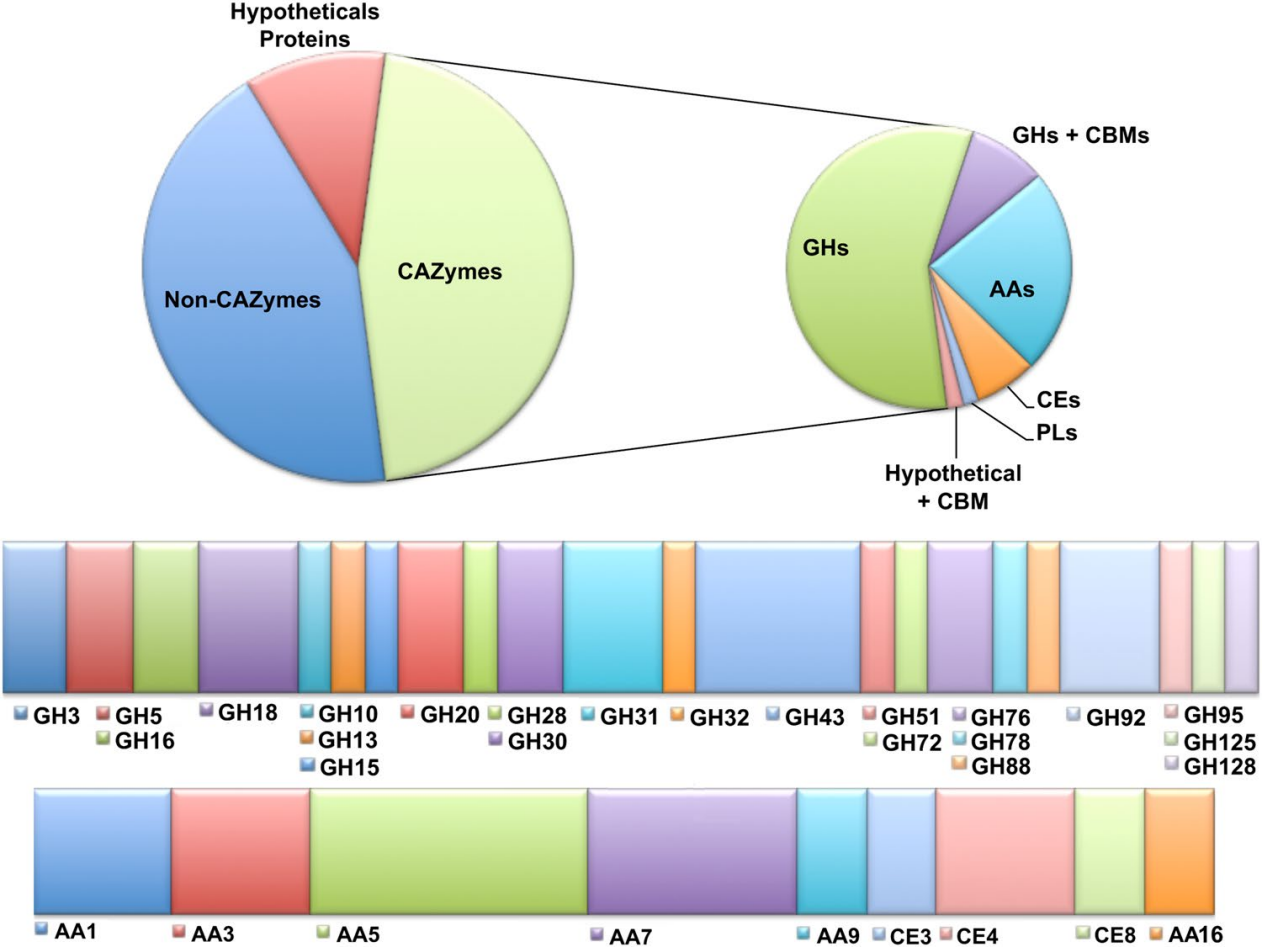
Distribution of proteins identified in the *Peniophora* sp. CBMAI 1063 secretome obtained after cultivation under saline conditions by mass spectrometry analysis (LC-MS/MS). Non-hypothetical proteins were classified into non-CAZymes and CAZymes groups.

Collectively, 126 proteins were identified in the secretome, of which 56 were classified as CAZymes, 57 were non-CAZymes and 13 were hypothetical proteins (Fig. 4, Table S2). As predicted by SignalP v.4.0^34^ and Yloc^35^, the majority of the enzymes identified (67%) in the secretome exhibited signal peptides. Among the ligninolytic enzymes, two laccases were identified which both presented predicted signal peptides. Although g15721.t1 presented the highest TPM value among all laccases in the transcriptome, the laccase g1591.t1 exhibited high spectrum counts (255 peptides) in comparison with all CAZymes identified in the secretome (Table 1). The laccase g1591.t1, herein named Pnh_Lac1, was identified with 12 unique peptides matches, covering 41% of the protein sequence. Four enzymes from family AA5 (related to glyoxal oxidases) were also identified in the secretome, all of which presented signal peptides. Peptides for peroxidases from family AA2, vanillyl-alcohol oxidases from AA4 and 1,4-benzoquinone reductase from AA6 were not found in the secretome, despite their relative abundance according to the genome and transcriptome data. However, this result should be considered expected since they lack peptide signals.

**Table 1:** Predicted lignin-active enzymes identified in the secretome of *Peniophora* sp. CBMAI 1063 cultivated in a bioreactor under saline conditions.

Accession Number	Molecular Weight^a^	Amino acid length^a^	dbCAN^b^	PFAM^c^	Description	Signal Peptide^d^	Location^e^	Unique peptides	Spectrum counts
**Lignin-Active Enzymes identified in the Secretome**									
g1591.t1	58 kDa	546	AA1	PF00394 PF07731 PF07732	Multicopper oxidase	YES	SP	12	255
g17194.t1	58 kDa	540	AA1	PF00394 PF07732 PF07731	Multicopper oxidase	YES	SP	3	2
g5706.t1	71 kDa	671	AA5	PF07250 PF09118	Glyoxal oxidase N- terminus	YES	SP	2	1
g5707.t1	81 kDa	769	AA5	PF07250 PF09118	Glyoxal oxidase N-terminus	YES	SP	3	1
g5709.t1	84 kDa	799	AA5	PF07250 PF09118	Glyoxal oxidase N-terminus	YES	SP	2	1
g9556.t1	59 kDa	554	AA5	PF07250 PF09118	Glyoxal oxidase N-terminus	YES	SP	4	5
g13672.t1	60 kDa	567	AA7	PF01565 PF08031	FAD binding domain berberine	YES	SP	5	6
g17067.t1	51 kDa	485	AA7	PF01565	FAD binding domain	YES	SP	3	5
g7475.t1	59 kDa	548	AA7	PF01565 PF08031	FAD binding domain berberine	YES	SP	3	1

^a^Molecular Weight and ^a^Amino acid length determined by LC-MS/MS. The results were processed by Mascot v.2.3.01 engine (Matrix Science Ltd.) software against the genome sequencing database of *Peniophora* sp. CBMAI and Scaffold – Proteome Software (version Scaffold_4.3.2 20140225). ^b^Web server and database for automated carbohydrate-active enzyme annotation generated based on the family classification from CAZy database: AA –Auxiliary Activity. ^c^Protein Family Domain analysis. ^d^The presence of a signal peptide of secreted proteins predicted by SignalP v.4.0. ^e^The subcellular localization of proteins predicted by YLoc (Interpretable Subcellular Localization Prediction): SP – secreted pathway; C – cytoplasm; M – mitochondrial location.

Among the cellulolytic and hemicellulolytic-active enzymes, the secretome contained 38 proteins classified in different families of GHs, which are involved in cellulose and hemicellulose breakdown (Tables 2–4). Enzymes from families GH6 and GH7 were absent in the secretome. Two GH5 were identified, where this family contains cellulase and hemicellulose activities. Five GHs exhibited CBMs: GH18 with CBM5, GH72 with CBM43, GH43 with CBM35, GH72 with CBM43 and GH15 with CBM20. Carbohydrate esterases (CE) from family 4 and 8, involved in deacetylation of hemicelluloses, and a polysaccharide lyase (PL) from family 22 (oligogalacturonate lyase) were also identified (Tables 3 and 5). Interestingly, a GH78 family whose characterized members are typically α-l-Rhamnosidases [E.C. 3.2.1.40] was identified along with a pectin-active enzymes group. These enzymes specifically cleave terminal α-l-rhamnose from a wide range of natural products and have important biotechnological applications in the food and pharmaceutical industries^53^.

**Table 2:** Predicted cellulose-active enzymes identified in the secretome of *Peniophora* sp. CBMAI 1063 cultivated in a bioreactor under saline conditions.

Accession Number	Molecular Weight^a^	Amino acid length^a^	dbCAN^b^	PFAM^c^	Description	Signal Peptide^d^	Location^e^	Unique peptides	Spectrum counts
**Cellulose-Active Enzymes**									
g13682.t1	71 kDa	640	AA3	PF05199 PF00732	Glucose-methanol-choline oxidoreductase	NO	C	18	19
g16244.t1	62 kDa	585	AA3	PF00732 PF05199	Glucose-methanol-choline oxidoreductase	NO	SP	6	7
g6504.t1	26 kDa	242	AA9	PF03443	Glycoside hydrolase family 61	YES	SP	3	71
g6425.t1	35 kDa	348	AA16	PF03067	Lytic polysaccharide monooxygenase	YES	SP	2	1
g11705.t1	81 kDa	760	GH3	PF01915 PF00933 PF14310	Glycoside hydrolase family 3	YES	SP	9	18
g15376.t1	93 kDa	879	GH3	PF01915 PF00933 PF14310	Glycoside hydrolase family 3	YES	SP	2	1
g1658.t1	37 kDa	353	GH5	PF00150	Glycoside hydrolase family 5	YES	SP	2	2
g5589.t1	49 kDa	452	GH5	PF00150	Glycoside hydrolase family 5	YES	SP	4	7

^a^Molecular Weight and ^a^Amino acid length determined by LC-MS/MS. The results were processed by Mascot v.2.3.01 engine (Matrix Science Ltd.) software against the genome sequencing database of *Peniophora* sp. CBMAI and Scaffold – Proteome Software (version Scaffold_4.3.2 20140225). ^b^Web server and database for automated carbohydrate-active enzyme annotation generated based on the family classification from CAZy database: GH- Glycoside Hydrolases; AA- Auxiliary Activities. ^c^Protein Family Domain analysis. ^d^The presence of a signal peptide of secreted proteins predicted by SignalP v.4.0. ^e^The subcellular localization of proteins predicted by YLoc (Interpretable Subcellular Localization Prediction): SP – secreted pathway; C – cytoplasm; M – mitochondrial location.

**Table 3:** Predicted hemicellulose-active enzymes identified in the secretome of *Peniophora* sp. CBMAI 1063 cultivated in a bioreactor under saline conditions.

Accession Number	Molecular Weight^a^	Amino acid length^a^	dbCAN^b^	PFAM^c^	Description	Signal Peptide^d^	Location^e^	Unique peptides	Spectrum counts
**Hemicellulose-Active Enzymes**									
g14451.t1	46 kDa	429	CE3	PF13472 PF00657	GDSL-like lipase/acyl hydrolase	YES	SP	7	44
g10047.t1	55 kDa	513	CE4		Carbohydrate esterase	YES	SP	3	5
g4642.t1	38 kDa	354	CE4	PF01522	Polysaccharide deacetylase	YES	SP	6	14
g11401.t1	37 kDa	345	GH10	PF00331	Glycoside hydrolase family 10	YES	SP	4	8
g11177.t1	59 kDa	540	GH125	PF06824	Protein of unknown function	NO	SP	5	4
g14331.t1	30 kDa	277	GH128	PF11790	Glycoside hydrolase catalytic core	YES	SP	2	6
g7518.t1	34 kDa	318	GH16		Glycoside hydrolases family 16	YES	SP	4	18
g7519.t1	33 kDa	313	GH16		Glycoside hydrolases family 16	YES	SP	6	66
g16729.t1	57 kDa	524	GH30	PF02055	Glycoside hydrolase family 30	YES	SP	3	3
g3624.t1	53 kDa	499	GH30	PF14587	O-Glycoside hydrolase family 30	YES	SP	10	57
g14525.t1	36 kDa	341	GH43	PF04616	Glycoside hydrolases family 43	YES	SP	2	2
g4370.t1	33 kDa	321	GH43	PF04616	Glycoside hydrolases family 43	YES	SP	4	54
g5765.t1	33 kDa	310	GH43	PF04616	Glycoside hydrolases family 43	YES	SP	5	55
g9420.t1	35 kDa	329	GH43	PF04616	Glycoside hydrolases family 43	YES	SP	2	13
g15433.t1	47 kDa	444	GH43 CBM35	PF04616	Glycoside hydrolases family 43	YES	SP	2	2
g3571.t1	69 kDa	638	GH51	PF06964	α-L-arabinofuranosidase	YES	SP	10	33
g14175.t1	58 kDa	564	GH72 CBM43	PF03198 PF07983	Glucanosyltransferase X8 domain	YES	SP	4	3
g11973.t1	40 kDa	375	GH76	PF03663	Glycoside hydrolase family 76	YES	SP	5	8
g4124.t1	40 kDa	370	GH76	PF03663	Glycoside hydrolase family 76	YES	SP	4	5
g11336.t1	91 kDa	827	GH92	PF07971	Glycoside hydrolase family 92	YES	SP	13	14
g1967.t1	67 kDa	606	GH92	PF07971	Glycoside hydrolase family 92	NO	SP	10	10
g5807.t1	88 kDa	812	GH92	PF07971	Glycoside hydrolase family 92	YES	SP	13	15
g5441.t1	186 kDa	1691	GH95	PF14498	Glycoside hydrolase family 95	NO	SP	2	1

^a^Molecular Weight and ^a^Amino acid length determined by LC-MS/MS. The results were processed by Mascot v.2.3.01 engine (Matrix Science Ltd.) software against the genome sequencing database of *Peniophora* sp. CBMAI and Scaffold – Proteome Software (version Scaffold_4.3.2 20140225). ^b^Web server and database for automated carbohydrate-active enzyme annotation generated based on the family classification from CAZy database: GH - Glycoside Hydrolases; CE - Carbohydrate Esterases; CBM: Carbohydrate-binding module. ^c^Protein Family Domain analysis. ^d^The presence of a signal peptide of secreted proteins predicted by SignalP v.4.0. ^e^The subcellular localization of proteins predicted by YLoc (Interpretable Subcellular Localization Prediction): SP – secreted pathway; C – cytoplasm; M – mitochondrial location.

**Table 4:** Predicted chitin, starch & other carbohydrate-active enzymes identified in the secretome of *Peniophora* sp. CBMAI 1063 cultivated in a bioreactor under saline conditions.

Accession Number	Molecular Weight^a^	Amino acid length^a^	dbCAN^b^	PFAM^c^	Description	Signal Peptide^d^	Location^e^	Unique peptides	Spectrum counts
**Chitin, Starch & Others Carbohydrate-Active Enzymes**									
g13668.t1	61 kDa	580	CBM20 GH15	PF00723 PF00686	Glycoside hydrolases family 15	YES	SP	20	197
g7315.t1	82 kDa	769	CBM5	PF17168 PF16335 PF08760 PF02839	Carbohydrate binding domain	YES	SP	10	15
g7861.t1	50 kDa	474	GH13	PF00128 PF02806	Alpha amylase	YES	SP	5	18
g11641.t1	97 kDa	890	GH31	PF01055 PF16863 PF13802	Glycoside hydrolases family 31	YES	SP	3	4
g15819.t1	109 kDa	997	GH31	PF01055 PF16863	Glycoside hydrolases family 31	NO	SP	12	9
g15820.t1	104 kDa	944	GH31	PF01055 PF16863	Glycoside hydrolases family 31	YES	SP	6	6
g11828.t1	60 kDa	547	GH32	PF00251	Glycoside hydrolases family 32	NO	SP	8	35
g11153.t1	49 kDa	460	GH88	PF07470	Glycoside hydrolase Family 88	YES	SP	3	3
g11942.t1	45 kDa	418	GH18	PF00704	Glycoside hydrolases family 18	YES	SP	2	12
g11425.t1	49 kDa	472	GH18; CBM5	PF02839	Carbohydrate binding domain	YES	SP	2	5
g6083.t1	136 kDa	1254	GH18; CBM5	PF00009 PF03764 PF14492 PF00679 PF00704 PF02839 PF03144	Elongation factor Tu GTP binding domain18	YES	M	7	23
g10368.t1	60 kDa	560	GH20	PF00728 PF14845	Glycoside hydrolase family 20	YES	SP	5	2
g15989.t1	134 kDa	1230	GH20	PF00728 PF14845 PF02838	Glycoside hydrolase family 20	YES	SP	7	9

^a^Molecular Weight and ^a^Amino acid length determined by LC-MS/MS. The results were processed by Mascot v.2.3.01 engine (Matrix Science Ltd.) software against the genome sequencing database of *Peniophora* sp. CBMAI and Scaffold – Proteome Software (version Scaffold_4.3.2 20140225). ^b^Web server and database for automated carbohydrate-active enzyme annotation generated based on the family classification from CAZy database: GH - Glycoside Hydrolases; CBM- Carbohydrate-binding module. ^c^Protein Family Domain analysis. ^d^The presence of a signal peptide of secreted proteins predicted by SignalP v.4.0. ^e^The subcellular localization of proteins predicted by YLoc (Interpretable Subcellular Localization Prediction): SP – secreted pathway; C – cytoplasm; M – mitochondrial location.

**Table 5:** Predicted pectin-active enzymes identified in the secretome of *Peniophora* sp. CBMAI 1063 cultivated in a bioreactor under saline conditions.

Accession Number	Molecular Weight^a^	Amino acid length^a^	dbCAN^b^	PFAM^c^	Description	Signal Peptide^d^	Location^e^	Unique peptides	Spectrum counts
**Pectin-Active Enzymes**									
g8265.t1	35 kDa	330	CE8	PF01095	Pectin esterase	YES	SP	2	2
g8087.t1	70 kDa	665	GH78	PF05592	Bacterial alpha-L-rhamnosidase	YES	SP	7	30
g10469.t1	74 kDa	673	PL22	PF07676	WD40-like beta propeller repeat	YES	SP	2	1
g1538.t1	45 kDa	413	GH28	PF00295	Glycoside hydrolase family 28	YES	SP	2	1

^a^Molecular Weight and ^a^Amino acid length determined by LC-MS/MS. The results were processed by Mascot v.2.3.01 engine (Matrix Science Ltd.) software against the genome sequencing database of *Peniophora* sp. CBMAI and Scaffold – Proteome Software (version Scaffold_4.3.2 20140225). ^b^Web server and database for automated carbohydrate-active enzyme annotation generated based on the family classification from CAZy database: GH- Glycoside Hydrolases; PL- Polysaccharide Lyases; CE- Carbohydrate Esterases. ^c^Protein Family Domain analysis. ^d^The presence of a signal peptide of secreted proteins predicted by SignalP v.4.0. ^e^The subcellular localization of proteins predicted by YLoc (Interpretable Subcellular Localization Prediction): SP – secreted pathway; C – cytoplasm; M – mitochondrial location.

### Omics data integration

Omics approaches can provide hypotheses regarding function for the large number of genes predicted from genome sequences. In this study, an integrative analysis of genomic, transcriptomic and proteomic data was performed for *Peniophora* sp. CBMAI 1063 Although the cultivation medium had the same composition, an important point to take in account is that the transcriptomic analysis was performed at the seventh day of small-scale cultivation (200 mL) while the secretome analysis was at the fifth day of cultivation in bioreactor scale (5 L). Moreover, it is well documented in the literature the poor correlation between transcriptomic and proteomic data due to several factors, including pre and pos translational processes^54,55^. Even though, omic data integration was performed in this work in order to explore the biotechnological potential of *Peniophora* sp. CBMAI 1063 under its optimized cultivation medium.

Thus, according to our omics data, of 18 genes encoding for laccases from family AA1 were found in the genome, only 2 proteins were identified as secreted in the condition analyzed (Figs. 3 and 4 and S1). Post-transcriptional, translational and degradation regulation events regarding the gene Pnh_Lac1 may be involved to explain the differences between gene expression and protein secretion under the conditions evaluated. Conversely, the fungus preferentially secrets Pnh_Lac1 in the optimized media (Fig. S1).

Family members from AA4 were not identified in the proteomic data, although 3 genes were identified in the genome and in the transcriptome (Figs. 3 and S2). This was similar to the observations for AA2, AA6 and AA8 family members, where the transcripts were expressed but the proteins were not identified in the secretome (Figs. 3 and 4). Regarding the family AA5, 4 proteins were found to be secreted from the 7 encoding genes identified in the genome (Figs. 3 and S2). Assigned to a glyoxal oxidase family domain according to PFAM, AA5 members are copper-containing enzymes that mainly oxidize aldehydes generated during lignin and carbohydrates degradation^56^. Although genes encoding glyoxal oxidases enzyme are widely distributed among white-rot fungi and symbiotic fungi, the number of characterized enzymes is still restricted^10^. The presence of these proteins in the secretome suggests that the fungus possesses a strong ligninolytic capacity. In this context, further studies of the AA5 enzymes and their role in *Peniophora* sp. CBMAI 1063 are extremely relevant. Furthermore, among the 27 genes encoding AA7 proteins, only 3 were found to be secreted.

**Figure 5:**
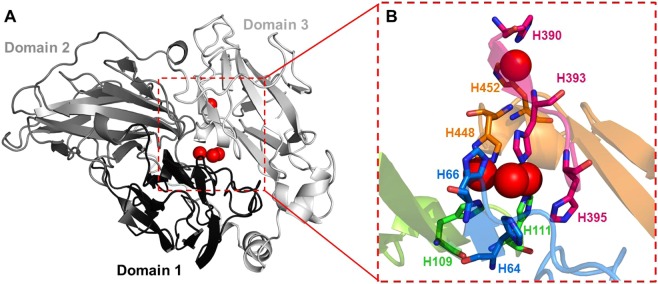
3D structure of Pnh_Lac1 generated by homology modeling. General structure of laccases, consisting of three cupredoxin domains (**A**) Zoom on the copper ion centers surrounded by the fungal laccase signature segments L1 (blue), L2 (green), L3 (magenta) and L4 (orange). The ion coordination is performed by the histidines presented in the L1-L4 regions (**B**).

According to the secretome data, in the case of the carbohydrate-binding module (CBM), predicted genes for CBM5, CBM20, CBM 21 and CBM 42 were found; while the transcriptomic data exhibited 4 transcripts for CBM21 and one for CBM20 (Figs. 3 and 4). Among all GH-coding genes identified, GH5 and GH43 exhibited the highest number of predicted genes (Fig. 3), and 2 and 5 proteins were identified in the secretome, respectively (Fig. 3).

### Homology and structural insights of the major laccase (Pnh_Lac1) secreted by *Peniophora* sp. CBMAI 1063

According to a sequence search using BLASTp, the most similar proteins to Pnh_Lac1 are multicopper oxidases from *Peniophora sp*. (87% identity to KZV66389.1 and 66% identity to KZV69698.1) and laccases from *P. lycii* (AWC08468.1) and *Meripilus giganteus* (CBV46340.1), both showing 61% identity. The laccase signature composed of four ungapped sequence segments L1–L4^57^ was in Pnh_Lac1 (Fig. S3). Segments L1-L4 contain the amino acids that bind to the copper centers: H64 and H66 from L1; H109 and H111 from L2; H390, H393 and H395 from L3; and H448 and H452 from L4 (Fig. 5B).

Performing a sequence search in the Protein Data Bank (PDB), the Pnh_Lac1 shows between 55–60% identity with different laccases from subfamily AA1_1. Due to the highly conserved structure of this family, the three-dimensional model of Pnh_Lac1 could be generated by homology modelling (Fig. 5), which was considered reliable according to the C-score value of 0.7 (range from −5 to 2)^37^. The enzyme presents typical folding of laccases, consisting of three cupredoxin domains with a mononuclear copper center located in domain 3, and a trinuclear center between domains 1 and 3^58^ (Fig. 5A). Collectively, these results, including the sequence alignment of the conserved regions, as well as the structural alignment of Pnh_Lac1 with other members of family AA1_1, are important to support the gene prediction data (Figs. 3 and S4) described in the present work.

### Pnh_Lac1 effects on lignin depolymerization and enzymatic digestibility of steam-exploded sugarcane bagasse

**Figure 6:**
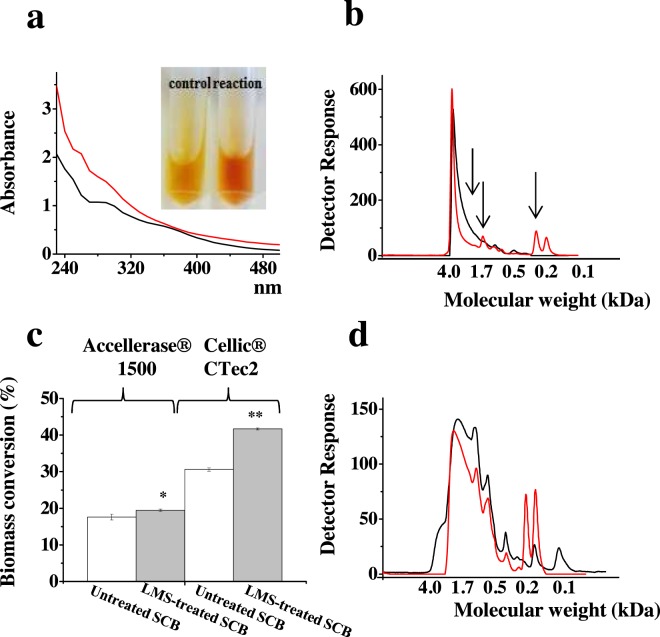
UV-light absorbance (**a**) and GPC chromatograms (**b**) showing the spectroscopic profile and molecular weight distribution of the lignin derived products obtained after the incubation of lignin extracted from SCB with purified Pnh_Lac1 and ABTS as the mediator (LMS); Enzymatic hydrolysis of LMS-treated SCB (grey bars) or non-treated SCB (white bars) performed with commercial cocktails at low dosage. The y-axis shows the cellulose conversion in percentage of the maximum theoretical cellulose conversion after 72 h at 50 °C. Error bars represent the standard errors of the means of triplicate experiments (**c**); GPC chromatograms showing the molecular weight distribution of the solubilized lignin-derived products and polyphenolics from LMS-treated SCB. Red lines (in figures **a**,**b**,**d**) represent experiments with LMS and black lines refer to control experiments without Pnh_Lac1.

The biochemical properties of purified Pnh_Lac1 were previously characterized by Mainardi *et al*.^2^: presenting 986.0 and 30.8 U mg^−1^, using ABTS and SGD as substrates, respectively, and optimum activity at pH 5.0 and 30 °C. To illustrate a potential biotechnological application, the purified Pnh_Lac1 was employed in combination with a synthetic mediator (ABTS) to promote the depolymerization of a lignin isolated from sugarcane bagasse by alkaline pretreatment^39^. The soluble fraction after incubation with LMS was analyzed by UV-light absorbance and GPC. An increase of 50% in UV-light absorbance at 280 nm was observed compared to the control experiment without Pnh_Lac1 (Fig. 6a). According to Arzola *et al*.^59^, spectra changes detected between 260 and 270 nm (in LMS-treated samples) indicate the introduction of new functional groups in the phenylpropane unit or aromatic rings of lignin fragments (Fig. 6a), resulting in auxochrome groups, new nonconjugated hydroxyl groups in the side-chain phenylpropane unit or new Cα–Cβ double bonds.

Spectral changes in the visible region were also detected, especially at 480 nm. Chromogen groups are commonly introduced after laccase activity, which is related to the reddish color appearance, as can be observed in Fig. 6a. The GPC analysis shows that soluble low molecular weight lignin derived compounds (<500 Da) were generated after LMS treatment (Fig. 6b), indicated by the arrows. The intense peak corresponding to high molecular weight (HMW) lignin (~4 kDa) appeared thinner when compared to the untreated sample, which is indicative of polymer depolymerization. The results indicate that Pnh_Lac1, when combined with a mediator, was able to promote lignin solubilization and depolymerization. This enzyme is therefore should be of biotechnological interest especially for the bioconversion of industrial lignins streams, including those generated from lignocellulose processing biorefineries where utilization is hampered by its chemically heterogeneous nature, low solubility and reactivity^38^.

Previously works have shown that LMS treatments can improve the enzymatic hydrolysis of lignocellulose materials, based on lignin solubilization, depolymerization and modification mechanisms^60^. However, there is also evidence that LMS can have detrimental effects on enzymatic hydrolysis yields, as a result of enzymes inhibition, oxygen competition and grafting of phenoxy radicals^39,61^. We are therefore led to believe that positive or negative effects can depend on singularities of the laccase employed and assay conditions.

In this sense, we evaluated the ability of the LMS based on Pnh_Lac1 and ABTS to remove lignin from pretreated SCB, which could improve the subsequent saccharification of lignocellulosic material. The SCB was first treated with LMS and then with two different commercial cocktails. Increases of 1.9% and 11.0% were observed in saccharification yield using Accellerase^®^ 1500 and Cellic^TM^ Ctec2, respectively, compared to the untreated samples (Fig. 6c). The GPC analysis of the soluble fraction after LMS treatment confirmed that the lignin fragments were partially degraded, leading to its solubilization into the supernatant (Fig. 6d).

The new peaks detected in the LMS-treated sample correspond to soluble low molecular weight phenolic compounds, between 400–200 Da (indicated by arrows) (Fig. 6). The HMW lignin fragments from 4–1.7 kDa were found in lower intensity in the chromatogram. Interesting, as can be observed in the LMS-treated supernatant, the peak corresponding to low molecular weight phenolic compounds (around 100 Da) is absent (Fig. 6).

The LPMO-containing cellulose cocktail Cellic™ Ctec2 has better performance than traditional enzyme cocktails mainly consisted of hydrolytic cellulases for biomass saccharification^62^. In addition, it has been demonstrated that soluble lignin derived compounds act as electron donors to LPMOs, boosting its catalytic efficiency^39,63^. Thus, the higher saccharification yield using Cellic™ Ctec2 for pretreated SCB may indicate that the soluble low molecular weight phenolic compounds generated by the LMS based on Pnh_Lac1 and ABTS enhanced the LPMO activity.

Collectively, our results demonstrate that Pnh_Lac1 could be applied be applied in bioconversion technologies, based on promoting lignin depolymerization and solubilization from the lignocellulosic substrate, thus facilitating the action of cellulolytic enzymes. However, further studies are necessary to combine the laccase with different mediators and higher enzyme loadings, which could improve the effectiveness of the LMS pre-treatment.

## Conclusions

For the first time, the genomic and secretomic analyses of marine-derived Peniophora sp. CBMAI 1063 revealed an important repertoire of valuable extracellular CAZymes, especially lignin and polyphenols-degrading enzymes. Interestingly, this marine fungus presents a higher number of unique orthologous gene clusters compared to the other two genomes from Peniophora species, demonstrating its singularity. In addition, our findings revealed that Peniophora sp. CBMAI 1063 has the ability to secrete a powerful set of oxidative enzymes of biotechnological interest, mainly the laccase Pnh_Lac1 which could be of interest in lignin modification and depolymerization strategies, bioconversion in industries and bioremediation.

## Supplementary information

SUPPLEMENTARY MATERIAL

## Data Availability

The authors promise the availability of supporting data.
