# Supplementary material for: Novel redox-active enzymes for ligninolytic applications revealed from multiomics analyses of Peniophora sp. CBMAI 1063, a laccase hyper-producer strain
Source: Sci Rep. 2019 Nov 26;9:17564. doi: 10.1038/s41598-019-53608-1 (PMC6879535; doi:10.1038/s41598-019-53608-1)
Supplement: Supplementary file 1 — SUPPLEMENTARY MATERIAL [file 41598_2019_53608_MOESM1_ESM.pdf]

SUPPLEMENTARY MATERIAL

Novel redox-active enzymes of ligninolytic applications revealed from multiomics analyses of Peniophora sp. CBMAI 1063, a laccase hyper-producer strain

Lívia B. Brenelli[a,b,d]†, Gabriela F. Persinoti[b]†, João Paulo L. Franco Cairo[e], Marcelo V. Liberato[e], Thiago Augusto Gonçalvez [e], Igor V. R. Otero[c], Pedro H. Mainardi[c], Claus Felby[d], Lara D. Sette[c] & Fabio M. Squina[e]*

[a] Instituto de Biologia, Universidade Estadual de Campinas (UNICAMP), Campinas, SP, Brazil.

[b] Laboratório Nacional de Ciência e Tecnologia do Bioetanol (CTBE), Centro Nacional de Pesquisa em Energia e Materiais (CNPEM), Campinas, SP, Brazil.

[c] Universidade Estadual Paulista (UNESP/RC/IB), Rio Claro, SP, Brazil.

[d] University of Copenhagen, Faculty of Science, Department of Geosciences and Natural Resource Management, Frederiksberg C, Denmark.

[e] Programa de Processos Tecnológicos e Ambientais, Universidade de Sorocaba, Sorocaba, SP, Brazil.

Figure S1. The aminoacid sequence of the laccase with the highest spectrum counts (g1591.t1). Peptides identified by the LC-MS/MS analysis are shown in bold red. The coverage for this protein was 41%

```
MSSSWSLLTL FSLVATGAFA AIGPTAQLTI TNANIAPDGL SRSSVLANGV
FPAPLITGNK GDAFSLTVAD SLTDTTMDLV TSIHWHGLFQ KSTNYADGVS
GVTQCPIVPD NSFEYAFSVP GQAGTFWYHS HYHAQYCDGL RGALVIYDPA
DPAASLYDVD DDSTVITLAD WYHYTSKNAP AIPAPSATLI NGLGRYSGGP
ASDLAVINVT KGTRYRFRLV SISCDTNFIF SIDNHKFSVI EVDGVNHKPQ
PIDNVQIFAG QRYSLVMTAD QDVGNYWVRA QPNNIAATFD GGLNSAILRY
KDATVADPTT TSSLSLALNE QDLHPLESVD YLSDKTPGGA DVNLELDVTF
TGGLFAVNGK SFEAPDVPVL LQILSGTPPA SLLPNGSVQL LPPNAVVEIA
IPGGVAAGPH PIHLHGHTFS VVRSAGNATY NYENPPIRDV VSIGTAATDR
TTIRFRTDNA GPWFMHCHID WHLTAGFAVV MAEDSEDVPN DVHPTDNWNA
LCPAWNTYSS TTGITQGGLK PIKAT
```

Figure S2.
Comparison of the genomic and secretomic CAZyme content of the marine-derived Peniophora sp. CBMAI 1

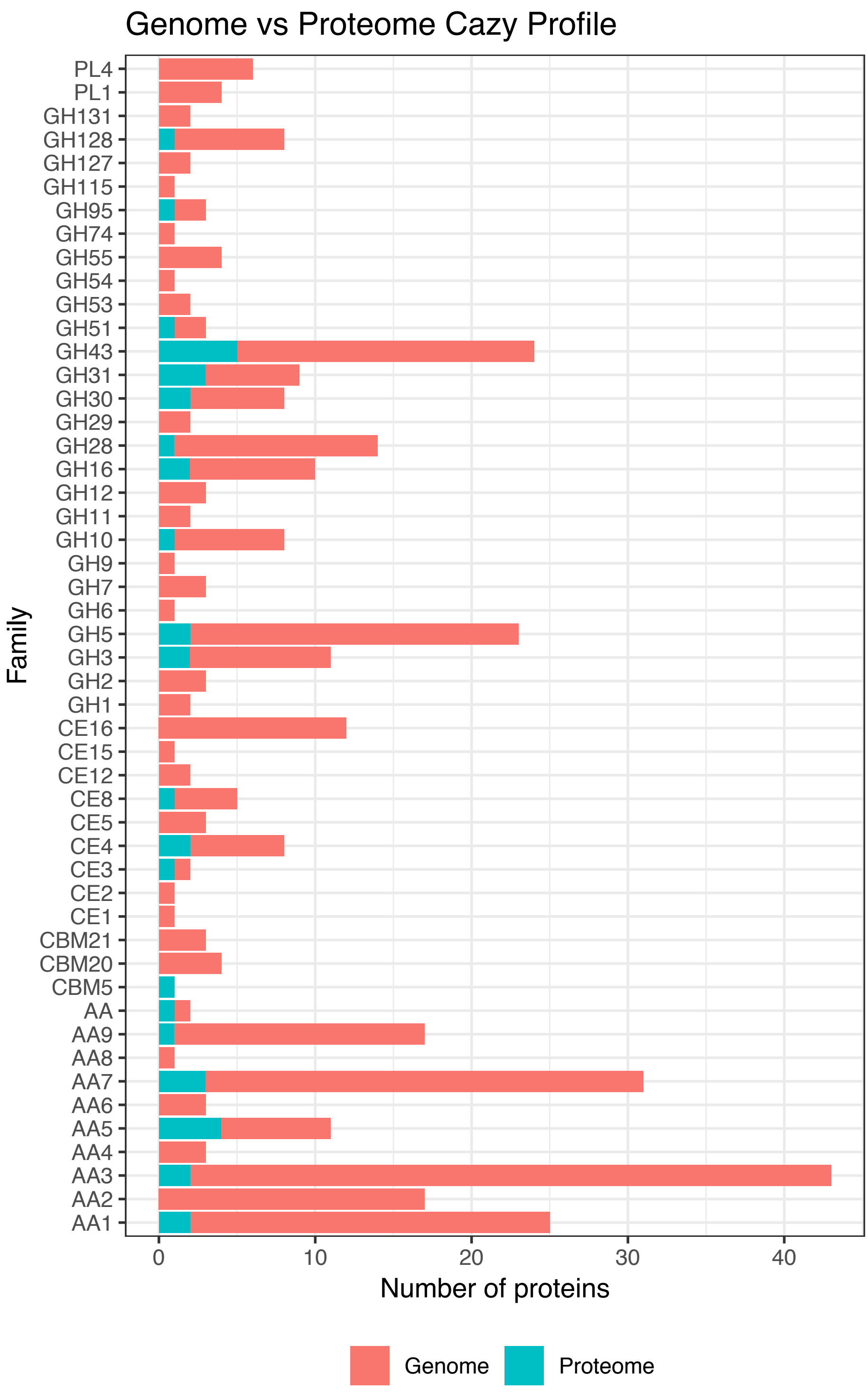

Figure S3. A Amino acid sequence alignment of Pnh_Lac1 with eight laccases from family AA1.1. Although the identity between Pnh_Lac1 with each is about 55 %, the fungal laccase signature segments, highlighted in blue (L1), green (L2), magenta (L3) and orange (4), showed high identity. Each laccase, except for Pnh_Lac1, is described by its genbank accession code.

```
Pnh_Lac1     MSSSWSLLTLFSL-VATGAFAAIGPTAQLTITNANIAPDGL-SRSSVLANGVFPAPLITG
 CAE81289    MPSFASLKSLVVLSLTSLSLAATVAL-DLHILNANLDPDGTGARSAVTAEGTTIAPLITG
 CAA78144    MAKFQSLLTFITLSLVASVYASIGPVADLTISNGAVSPDGF-SRQAILVNDVFPSPLITG
 ALE66817    MA-FKLTVSFLLLGLSLSARAAIGPVADLKIVNANIQPDGF-TRPAVLAGGTFPGPLIKG
 AIT51846    MKALSFLTPIVTLALVAGAFASVGPVANLKIGNAAVSPDGY-TRDAVVVNGATPGPLIVG
 AGE13770    MAGFQTLLAYITLSLVASVYAGIGPVTDLTISDGPVSPDGF-TRQAILVNNQFPSPLITG
 AFI41889    MASFKSIAALIAL-IFAVNAAQIGPVTDLHITNANISPDGF-SRPAVLAGGTFPGPTIAG
 AF118267    M--FKNLLSFALLAISVANAQIVNSVDTMTLTNANVSPDGF-TRAGILVNGVH-GPLIRG
 ACC43989    MSRFQSLLTFINISLVAVAHAAVGPVADLTITDAAVSPDGF-SRQAVVVNGVTPGPLVAG

Pnh_Lac1     NKGDAFSLTVADSLTDTTMDLVTSIHWHGLFQKSTNYADGVSGVTQCPIVPDNSFEYAFS
 CAE81289    NIDDRFQINVIDQLTDANMRRATSIHWHGFFQAGTTEMDGPAFVNQCPIIPNESFVYDFV
 CAA78144    NKGDRFQLNVIDNMTNHTMLKSTSIHWHGFFQHGTNWADGPAFVNQCPISTGHAFLYDFQ
 ALE66817    NKGDNFQLNVIDELENEDMLKSTSIHWHGFFQHGTNWADGPAFVNQCPITTGHSFLYNFH
 AIT51846    NKGDNFRLNVIDELTNHTMLKSTSIHWHGFFQHGTNWADGGAFVNQCPISSGHSFSYNFQ
 AGE13770    NKGDRFQLNVIDNMNNHTMLKSTSIHWHGFFQHGTNWADGPAFVNQCPISPGHSFLYDFQ
 AFI41889    NTGDNFQITVFNDLTDPSMLTDTSIHWHGLFQKGTNWADGPAFVTQCPIITGQSFDYNFN
 AF118267    GKNDNFELNVVNDLDNPTMLRPTSIHWHGLFQRGTNWADGADGVNQCPISPGHAFLYKFT
 ACC43989    NIGDRFQLNVIDNLTNHTMLKSTSIHWHGFFQHGTNWADGPAFINQCPISPGHSFLYDFQ

Pnh_Lac1     VPGQAGTFWYHSHYHAQYCDGLRGALVIYDPADPAASLYDVDDDSTVITLADWYHYTSK-
 CAE81289    VPGQAGTYWYHSHLSTQYCDGLRGAFVVYDPNDPHLSLYDVDDASTVITIADWYHSLSTV
 CAA78144    VPDQAGTFWYHSHLSTQYCDGLRGPIVVYDPQDPHKSLYDVDDDSTVITLADWYHLAAKV
 ALE66817    VPDQAGTFWYHSHLSTQYCDGLRGPMVVYDPHDPLKQLYDVDDESTVMTLADWYHTLARQ
 AIT51846    AKDQAGTFWYHSHLSTQYCDGLRGPFVVYDPKDPHKKLYDVDNESTVITLEDWYHTAARL
 AGE13770    VPDQAGTFWYHSHLSTQYCDGLRGPIVVYDPQDPHKDLYDVDDDSTVITLADWYHLAAKV
 AFI41889    VPGQAGTFWYHSHLSTQYCDGLRGPFVVYDPNDPNASLYDVDDDTTIITLADWYHTLAQQ
 AF118267    PAGHAGTFWYHSHFGTQYCDGLRGPMVIYDDNDPHAALYDEDDENTIITLADWYHIPAP-
 ACC43989    VPDQAGTFWYHSHLSTQYCDGLRGPFVVYDPNDPHASRYDVDNDDTVITLADWYHTAAKL

Pnh_Lac1     ----NAPAIPAPSATLINGLGRYSGGPA-SDLAVINVTKGTRYRFRLVSISCDTNFIFSI
 CAE81289    LFPNPNKAPPAPDTTLINGLGRNSANPSAGQLAVVSVQSGKRYRFRIVSTSCFPNYAFSI
 CAA78144    -----GPAVPTADATLINGLGRSI-NTLNADLAVITVTKGKRYRFRLVSLSCDPNHTFSI
 ALE66817    ----EPPGPVTPDSTLINGLGRAPGQTTPSELAVLTVKRGTRYRIRLINISCEPNYHYSI
 AIT51846    ----GPRFPLGSDSTLINGLGRSA-TTATGDLAVIKVTRGKRYRFRLVSLSCDPFYTFSI
 AGE13770    -----GPAVPTADATLINGLGRSI-STLNADLAVISVTKGKRYRFRLVSLSCDPNHTFSI
 AFI41889    ---EPIGAAITADATLINGLGRSFTNTTASPLSVITVQSGKRYRMRLVSISCDPNYLFSI
 AF118267    ----SIQGAAQPDATLINGKGRYVGGPA-AELSIVNVEQGKKYRMRLISLSCDPNWQFSI
 ACC43989    ----GPRFPGGADATLINGKGRAP-SDSPAELSVIKVTKGKRYRFRLVSLSCNPNHTFSI

Pnh_Lac1     DNHKFSVIEVDGVNHKPQPIDNVQIFAGQRYSLVMTADQDVGNYWVRAQPNNIA----AT
 CAE81289    DGHRMTVIEVDGVSHQPLTVDSLTIFAGQRYSVVVEANQAVGNYWIRANPSNGRN----G
 CAA78144    DGHSLTVIEADSVNLKPQTVDSIQIFAAQRYSFVLNADQDVDNYWIRALPNSGT----RN
 ALE66817    DNHDLTVIEADGVSTQSLTVSSLTIFAGQRYSFILNANQPVGNYWIRAQPNDAAD---VT
 AIT51846    DGHNMTIIEADAVNTKPHTVDSLEIFAGQRYSFILNANQPVDNYWVRANPNFGN----VG
 AGE13770    DGHTMTVIEADSVNLKPQVVDSIQIFAAQRYSFVLNADQDIGNYWIRAMPNSGT----RN
 AFI41889    DGHDMTIIEVDGVNSQQLTVDQIQIFAAQRYSFVLNANQPVGNYWIRAQPNSGG----QG
 AF118267    DGHELTIIEVDGQLTEPHTVDRLQIFTGQRYSFVLDANQPVDNYWIRAQPNKGRNGLAGT
 ACC43989    DGHNLTIIEVDSVNSQPLEVDSIQIFAAQRYSFVLDANQAVDNYWIRANPNFGN----VG
```

```
Pnh_Lac1     FDGGLNSAILRYKDATVADPTTT--SSLSLALNEQDLHPLESVDYLSDKTPGGADVNLEL
 CAE81289    FTGGINSAIFRYEGAAVAEPTTS--QNSGTALNEANLIPLINPGAPGNPVPGGADINLNL
 CAA78144    FDGGVNSAILRYDGAAPVEPTTT-QTPSTQPLVESALTTLEGTAAPGNPTPGGVDLALNM
 ALE66817    FNGGINSAILRYEGAPVAEPNTT-AGPDNTPLLEVNIRPFVFTPVPGQPHAGGADFVKNL
 AIT51846    FTNGINSAILRYDGAAVAEPATAIPPASVTPLLETDLHPLVSTPVPGSPVAGGVDKALNF
 AGE13770    FDGGVNSAILRYDGADPVEPTTT-QTPSSQPLVESALTTLEGTAAPGSPTAGGVDLAINM
 AFI41889    FDGGINSAILRYEGATVEDPTTTAPTTFSNPLVETDLHPLADLGVPGQPFRGGADDPLVL
 AF118267    FANGVNSAILRYAGAANADPTTS-ANPNPAQLNEADLHALIDPAAPGIPTPGAADVNLRF
 ACC43989    FDGGINSAILRYDGAPAVEPTTN-QTTSVKPLNEVDLHPLVSTPVPGSPSSGGVDKAINM

Pnh_Lac1     DVTFTGG-L-FAVNGKSFEAPDVPVLLQILSGTPPAS-LLPNGSVQLLPPNAVVEIAIPG
 CAE81289    RIGRNATTADFTINGAPFIPPTVPVLLQILSGVTNPNDLLPGGAVISLPANQVIEISIPG
 CAA78144    AFGFAGG--RFTINGASFTPPTVPVLLQILSGAQSAQDLLPSGSVYSLPANADIEISLPA
 ALE66817    LFSFNGT--NFQVDNVSFVPPTVPILLQILSGAHTAQDLMPAGSIIPLPKNAVIEFSMPG
 AIT51846    VFNFDGT--NFFINDATFTPPSVPVLLQILSGAQAAQDLLPSGSVIPLPALSTIELSFPA
 AGE13770    AFGFAGG--RFTINGASFTPPTVPVLLQILSGAQNAQDLLPTGSVYSLPANADIEISLPA
 AFI41889    NLAFANG--RFSIDGVSFVPPTVPVLLQILSGAQNAQDLLPAGSVISLPSNSVIEVALPA
 AF118267    QLGFSGG--RFTINGTAYESPSVPTLLQIMSGAQSANDLLPAGSVYELPRNQVVELVVPA
 ACC43989    AFNFNGS--NFFINGASFVPPTVPVLLQILSGAQTAQDLLPSGSVYVLPSNASIEISFPA

Pnh_Lac1     GVA--AGPHPIHLHGHTFSVVRSAGNATYNYENPPIRDVVSIGTA-ATDRTTIRFRTDNA
 CAE81289    -----GGNHPFHLHGHNFDVVRTPGSSVYNYVNPVRRDVVSIGGG--GDNVTFRFVTDNP
 CAA78144    TSAAPGFPHPFHLHGHTFAVVRSAGSSTYNYANPVYRDVVSTGSP--GDNVTIRFRTDNP
 ALE66817    GVV--GGGHPIHLHGHNFWVIRSANSSVYNYNDPVIRDVVNIGTT--GDNVTIRFETNNP
 AIT51846    TANAPGVPHPFHLHGHTFAVVRSAGSTAYNYEDPVWRDVVSTGTPAAGDNVTIRFVTDNP
 AGE13770    TAAAPGFPHPFHLHGHTFAVVRSAGSSTYNYANPVYRDVVSTGSP--GDNVTIRFRTDNP
 AFI41889    GAA--GGPHPFHLHGHNFAVVQSANNATPNYVNPIWRDTVSIGGT--GDNVTIRFTTNNP
 AF118267    GVL--GGPHPFHLHGHAFSVVRSAGSSTYNFVNPVKRDVVSLGVT--GDEVTIRFVTDNP
 ACC43989    TAAAPGAPHPFHLHGHTFAVVRSAGSTVYNYDNPIFRDVVSTGTPAAGDNVTIRFDTNNP

Pnh_Lac1     GPWFMHCHIDWHLTAGFAVVMAEDSEDVPNDVHPTDNWNALCPAWN-----TYSS-----
 CAE81289    GPWFLHCHIDWHLEAGLAVVFAEDIPNIPIANAISPAWDDLCPKYN-----ANN------
 CAA78144    GPWFLHCHIDFHLEAGFAVVMAEDIPDVAATNPVPQAWSDLCPTYD-----ALS------
 ALE66817    GPWFLHCHIDWHLDLGFAVVMAEDIPDAAAANPVPAAWNELCPLYD-----ALT------
 AIT51846    GPWFLHCHIDFHLEAGFAVVFAEDLPGTPAANPVPQSWSDLCPIYD-----ALA------
 AGE13770    GPWFLHCHIDFHLEAGFAVVMAEDIPDVAAVNPVPQAWSDLCPTYN-----ALD------
 AFI41889    GPWFLHCHIDWHLEAGFAIVFAEDIPDTASANPVPQAWSDLCPAYDQAHNISTATRQDFQ
 AF118267    GPWFFHCHIEFHLMNGLAIVFAEDMANTVDANNPPVEWAQLCEIYDDLPPEATSIQT---
 ACC43989    GPWFLHCHIDFHLEGGFAVVMAEDTPDVKAVNPVPQAWSDLCPTYD-----ALD------

Pnh_Lac1     ---TTGITQGGLKP---IKAT
 CAE81289    -------PDSGLA--------
 CAA78144    -------PDDQ----------
 ALE66817    -------PGNQ----------
 AIT51846    -------EDDQ----------
 AGE13770    -------PNDQ----------
 AFI41889    ILCICGILHVNFRQEERCGIS
 AF118267    --VVRRAEPTGFSAKFRREGL
 ACC43989    -------PNDQ----------
```

Figure S4. Structural alignment of Pnh_Lac1 model with the structures of the eight laccases from family AA1.1, used in the sequence alignment described in figure S2. The Pnh_Lac1 structure, generated by homology modelling, resulted in a high structural identity (RMSD about 0.5 Å) with the other members, evi66dencing the conserved folding of the family and the reliability of the model.

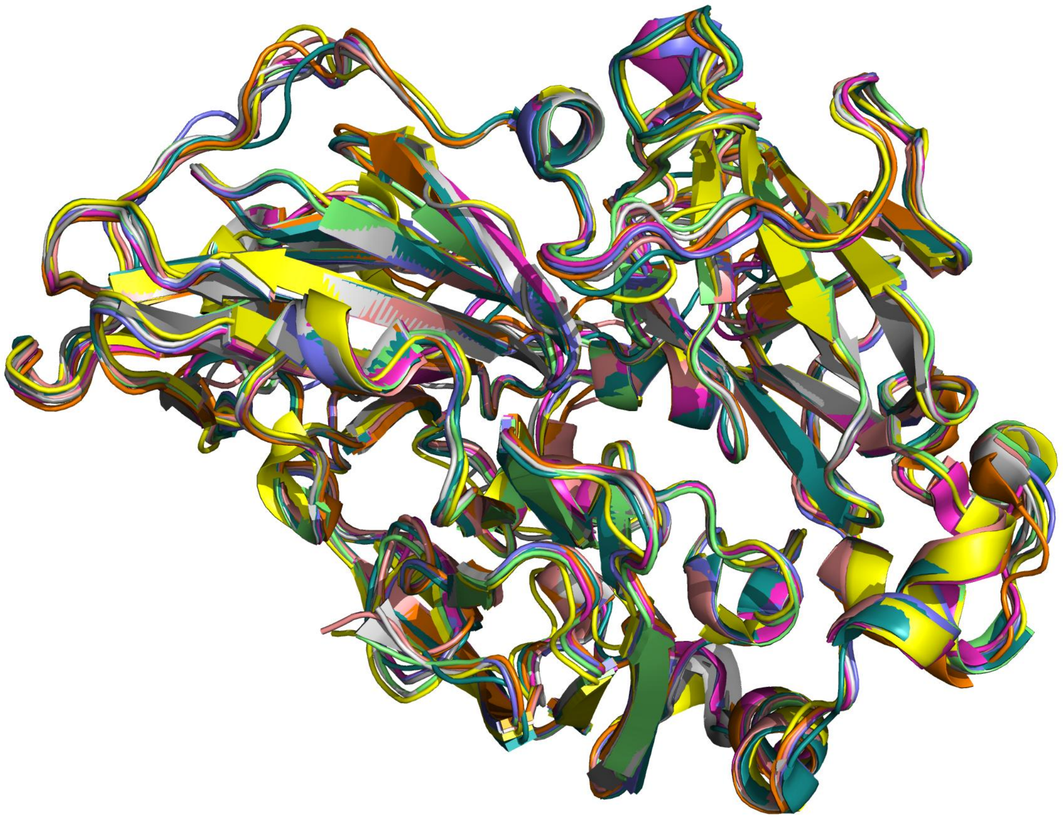

Table S1. Statistics of the marine *Peniophora* sp. CBMAI 1063 draft genome assembly.

Assembly statistics	*Peniophora* sp. CBMAI 1063
# Number of QC reads	39442518
Estimated coverage	165X
# contigs (>= 0 bp)	939
# scaffolds	833
# contigs (>= 5000 bp)	618
# contigs (>= 10000 bp)	509
# contigs (>= 25000 bp)	376
# contigs (>= 50000 bp)	249
Total length (bp)	46982497
Largest contig (bp)	1115204
GC (%)	55,21
N50	155805
N75	73455
L50	84
L75	191
Number of predicted genes	17219
Number of predicted transcripts	17714
Average gene length (bp)	1416
rRNA	1
tRNA	74
Genome completeness (%)	93

Table S2. Non-CAZymes identified in the secretome of *Peniophora* sp. CBMAI 1063 cultivated in a bioreactor under saline conditions.

Accession Number	Molecular Weight[a]	Amino acid length[b]	PFAM Domain[c]	PFAM description	Signal Peptide[d]	Location[e]	Unique peptides	Spectrum counts
g1019.t1	34 kDa	305	PF00240<br>PF11976<br>F14560<br>PF13881	Ubiquitin family	NO	C	2	43
g10257.t1	42 kDa	394	PF01161	Phosphatidylethanolamine-binding protein	NO	SP	2	1
g6425.t1	35 kDa	348	PF03067	Lytic polysaccharide monooxygenase	YES	SP	2	1
g10717.t1	45 kDa	424	PF00026<br>PF14543	Eukaryotic aspartyl protease<br>Xylanase inhibitor N-terminal	YES	SP	3	3
g11007.t1	36 kDa	321	PF00248	Aldo/keto reductase family	NO	C	3	9
g11209.t1	44 kDa	393	PF04389<br>PF01546	Peptidase family M28/ M20/M25/M40	YES	SP	5	11
g11268.t1	120 kDa	1084	PF00282	Pyridoxal-dependent decarboxylase conserved domain	NO	C	15	20
g1175.t1	29 kDa	258	PF01182	Glucosamine-6-phosphate isomerases<br>6-phosphogluconolactonase	NO	C	2	9
g12242.t1	53 kDa	505	PF07992<br>PF02852<br>PF00070<br>PF12831<br>PF01134<br>PF00890	Pyridine nucleotide-disulphide oxidoreductase;<br>FAD dependent oxidoreductase:	NO	M	8	10
g1234.t1	31 kDa	279	PF01738	Dienelactone hydrolase Family	NO	M	4	13
g12438.t1	14 kDa	135	PF07249	Cerato-platanin	YES	SP	3	29
g12440.t1	14 kDa	135	PF07249	Cerato-platanin	YES	SP	2	26

g1251.t1	39 kDa	397	not identified		YES	SP	3	8
g12536.t1	166 kDa	1524	PF00168	C2 domain	NO	X	4	9
g13042.t1	43 kDa	395	PF06516	Purine nucleoside permease (NUP)	NO	SP	4	13
g1333.t1	61 kDa	561	PF02878 PF02880 PF02879	Phosphoglucomutase/ phosphomannomutase, alpha/beta/alpha domain I	NO	C	3	4
g13681.t1	87 kDa	793	not identified		NO	C	4	2
g13797.t1	39 kDa	379	not identified		YES	SP	3	58
g13878.t1	52 kDa	479	PF04389	Peptidase family M28	YES	SP	4	7
g14070.t1	26 kDa	231	PF13417 PF13409 PF02798	Glutathione S-transferase	NO	M	2	6
g14182.t1	21 kDa	200	not identified		YES	SP	2	7
g14619.t1	61 kDa	548	PF00262	Calreticulin Family	YES	SP	2	3
g14686.t1	38 kDa	358	PF10282	Lactonase, 7-bladed beta-propeller	NO	SP	4	3
g14843.t1	22 kDa	198	not identified		NO	M	3	43
g14865.t1	34 kDa	317	PF01014 PF01014	Uricase;Uricase	NO	M	3	5
g15561.t1	38 kDa	374	not identified		YES	SP	3	5
g16265.t1	47 kDa	438	PF01546 PF07687	Peptidase family M20/M25/M40	NO	C	2	1
g16319.t1	26 kDa	253	PF00314	Thaumatin Family	YES	SP	3	9

g16410.t1	17 kDa	152	PF00334	Nucleoside diphosphate kinase	NO	M	2	7
g16808.t1	26 kDa	236	PF13419<br>PF00702<br>PF13242	Haloacid dehalogenase-like hydrolase	NO	C	2	6
g16849.t1	67 kDa	617	PF09423<br>PF16655	PhoD-like phosphatase	YES	SP	7	10
g1891.t1	27 kDa	256	PF00314	Thaumatin Family	YES	SP	3	18
g312.t1	62 kDa	581	PF12222	Peptide N-acetyl-beta-D-glucosaminyl asparaginase amidase A	YES	SP	2	4
g329.t1	76 kDa	696	PF03571	Peptidase family M49	NO	C	16	13
g3731.t1	54 kDa	497	PF01266	FAD dependent oxidoreductase	YES	SP	2	1
g4046.t1	33 kDa	305	not identified		YES	SP	3	2
g414.t1	35 kDa	348	PF03330	Lytic transglycolase	YES	SP	2	1
g4288.t1	35 kDa	322	PF03372	Endonuclease/Exonuclease/phosphatase family	NO	C	11	68
g514.t1	41 kDa	396	not identified		YES	SP	6	25
g5214.t1	61 kDa	568	PF00026	Eukaryotic aspartyl protease	YES	SP	2	1
g5591.t1	55 kDa	515	PF00883<br>PF02789	Cytosol aminopeptidase family, catalytic domain	NO	M	2	1
g5969.t1	11 kDa	99	PF11976<br>PF00240	Ubiquitin-2 like Rad60 SUMO-like	NO	N	2	7
g6000.t1	19 kDa	168	PF14566	Inositol hexakisphosphate	NO	C	3	14
g620.t1	44 kDa	405	PF08450<br>PF03088	SMP-30/Gluconolaconase/LRE-like region;Strictosidine synthase	YES	M	4	4

g6204.t1	36 kDa	343	not identified		YES	SP	2	2
g621.t1	44 kDa	409	PF08450 PF03088	SMP-30/Gluconolaconase/LRE-like region;Strictosidine synthase	YES	SP	3	2
g690.t1	97 kDa	905	PF13449 F01266	Esterase-like activity of phytase;FAD dependent oxidoreductase	YES	SP	18	42
g7075.t1	24 kDa	227	not identified		YES	SP	4	13
g7191.t1	58 kDa	529	PF05577	Serine carboxypeptidase S28	YES	SP	5	7
g7314.t1	74 kDa	691	PF17168 PF16335 PF08760	Domains of unknown function	YES	SP	6	13
g7338.t1	74 kDa	687	PF03372	Endonuclease/Exonuclease/phosphatase family	NO	C	2	2
g7407.t1	106 kDa	960	PF04253 PF04389 PF02225	Transferrin receptor-like dimerisation domain; Peptidase family M28;PA domain	NO	N	5	8
g7610.t1	68 kDa	615	PF00149	Calcineurin-like phosphoesterase	YES	SP	3	2
g7664.t1	39 kDa	403	not identified		YES	SP	4	5
g780.t1	37 kDa	338	PF07991 PF01450 PF03807 PF03446	Acetohydroxy acid isomeroreductase, NADPH-binding domain	NO	C	4	6
g7893.t1	63 kDa	590	PF09286 PF00082		YES	SP	8	13

g8153.t1	51 kDa	470	PF07992<br>PF02852<br>PF00070<br>PF13738<br>PF03486<br>PF13434	Pyridine nucleotide-disulphide oxidoreductase	NO	C	10	16
g8392.t1	102 kDa	934	not identified		NO	SP	7	6
g8584.t1	87 kDa	774	PF01432	Peptidase family M3	NO	SP	4	3
g8812.t1	100 kDa	903	PF01433<br>PF11838	Peptidase family M1;ERAP1-like C-terminal domain	NO	C	6	3
g8977.t1	64 kDa	580	PF05577	Serine carboxypeptidase S28	NO	C	3	2
g9286.t1	33 kDa	308	not identified		YES	SP	2	2
g9390.t1	44 kDa	427	PF00445	Ribonuclease T2 family	YES	SP	6	28
g9486.t1	42 kDa	389	PF01263	Aldose 1-epimerase	YES	SP	3	3
g9536.t1	50 kDa	466	PF00557	Metallopeptidase family M24	NO	N	5	9
g9683.t1	14 kDa	125	PF00173	Cytochrome b5-like Heme/Steroid binding domain	NO	M	2	7
g1396.t1	58 kDa	541	PF00135<br>PF07859	Carboxylesterase family 10	YES	SP	2	3
g1415.t1	58 kDa	541	PF00135<br>PF07859	Carboxylesterase family 10	YES	SP	4	5
g1747.t1	59 kDa	549	PF00135<br>PF07859	Carboxylesterase family 10	YES	SP	3	4
g5988.t1	63 kDa	584	PF00135	Carboxylesterase family 10	NO	SP	8	8

Legend. [a]Molecular Weight and [b]Amino acid length determined by LC-MS/MS analysis . The results were processed by Mascot v. 2.3.01 engine (Matrix Science Ltd.) software against the genome sequencing database of *Peniophora* sp. CBMAI 1063 and Scaffold – Proteome Software (version Scaffold_4.3.2 20140225). [c] Pfam protein family Domain analysis. [d]The presence of a signal peptide of secreted proteins predicted by SignalP v.4.0. [e]The subcellular localization of proteins predicted by YLoc (Interpretable Subcellular Localization Prediction): SP – secreted pathway; C – cytoplasm; M – mitochondrial location; N – Nucleus
